# Shikonin Ameliorates Alcohol‐Associated Liver Disease by Reducing Oxidative Stress, Apoptosis, and Inflammation

**DOI:** 10.1002/fsn3.71064

**Published:** 2025-10-08

**Authors:** Haining Gan, Miaoling Gong, Lingzhi Wang, Xuejun Huang, Yuxing Chen, Zixuan Hu, Xuewen Li, Qiaohuang Zeng

**Affiliations:** ^1^ Guangdong Provincial Second Hospital of Traditional Chinese Medicine (Guangdong Provincial Engineering Technology Research Institute of Traditional Chinese Medicine) Guangzhou China; ^2^ Guangdong Provincial Key Laboratory of Research and Development in Traditional Chinese Medicine Guangzhou China; ^3^ The Fifth Clinical College of Guangzhou University of Chinese Medicine Guangzhou China; ^4^ Center for Drug Research and Development Guangdong Pharmaceutical University Guangzhou Guangdong China; ^5^ School of Traditional Chinese Medicine and Health Nanfang College Guangzhou China

**Keywords:** alcoholic liver disease (ALD), anti‐apoptosis, anti‐inflammatory, antioxidant, shikonin (SKN)

## Abstract

Alcohol‐associated liver disease (ALD) represents a major global health concern due to its significant contribution to morbidity and mortality. The development of ALD is largely driven by oxidative stress, inflammation, and apoptosis. Shikonin (SKN) is a natural extract possessing both antioxidant and anti‐inflammatory properties. This study investigated the protective effects of SKN against ALD in an in vivo rat model of ALD induced by daily gavage with 52% alcohol for 6 weeks. Following this, SKN was administered, and its antioxidant, anti‐inflammatory, and anti‐apoptotic properties were examined. Results demonstrated that SKN treatment inhibited the alcohol‐induced increase of alanine aminotransferase (ALT), aspartate aminotransferase (AST), alkaline phosphatase (ALP), total cholesterol (TC), triglycerides (TG), lipopolysaccharide (LPS), reactive oxygen species (ROS), interleukin‐1β (IL‐1β), tumor necrosis factor‐α (TNF‐α), and interleukin‐6 (IL‐6) in serum and liver tissue, while enhancing the anti‐inflammatory factor IL‐10. Moreover, SKN mitigated alcohol‐induced histological changes in liver tissues. In addition, the administration of SKN led to the restoration of hepatic superoxide dismutase (SOD) levels while reducing malondialdehyde (MDA) levels. Western blot revealed that SKN notably decreased Keap1 expression and elevated the expression of Nrf2 and HO‐1 proteins, thereby enhancing the antioxidant capacity. In addition, SKN mitigated inflammation by inhibiting the TLR4/MyD88/NF‐κB pathway. Furthermore, SKN administration inhibited the expression of Bax and Caspase3, while upregulating the anti‐apoptotic protein Bcl‐2, thereby reducing hepatocyte injury through the inhibition of apoptosis. Overall, these results indicated that SKN could ameliorate ALD by mitigating oxidative stress, apoptosis, and inflammation, positioning it as a promising natural extract for preventing and treating ALD.

## Graphical abstract

This research indicated that SKN modulated the Keap1/Nrf2/HO‐1 pathway to activate the antioxidant defense system and alleviate oxidative stress. It also suppresses inflammation by downregulating the TLR4/MyD88/NF‐κB signaling pathway and prevents hepatocyte apoptosis by regulating the Bax/Bcl‐2/Caspase‐3 signaling pathway. Our findings indicate that SKN could be a promising therapeutic agent for ALD.

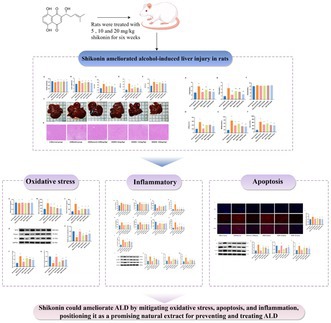

**Abbreviations**
ADH: alcohol dehydrogenase
ALD: alcoholic liver disease
ALDH: aldehyde dehydrogenase
ALP: alkaline phosphatase
ALT: alanine transaminase
AST: aspartate transaminase
Bax: BCL2‐associated X protein
Bcl‐2: B‐cell lymphoma 2
BUN: blood urea nitrogen
Caspase‐3: cysteine‐aspartic acid protease 3
Con A: concanavalin A
CRE: creatinine
ECL: enhanced chemiluminescence
GLU: glucose
HE: hematoxylin–eosin
HO‐1: nuclear factor erythroid 2‐related factor 2
IFN‐γ: interferon‐gamma
IL‐17A: interleukin‐17A
IL‐1β: interleukin‐1β
IL‐6: interleukin‐6
JNK: C‐Jun N‐terminal kinase
Keap1: Kelch‐like ECH‐associated protein 1
LPS: lipopolysaccharide
MDA: malondialdehyde
MyD88: toll‐like receptor 4
NF‐κB: nuclear factor kappa‐light‐chain‐enhancer of activated B cells
Nrf2: nuclear factor erythroid 2‐related factor 2
ROS: reactive oxygen species
SKN: shikonin
SOD: superoxide dismutase
TC: cholesterol
TG: triglyceride
TLR4: toll‐like receptor 4
TNF‐α: tumor necrosis factor‐α
UA: uric acid
WHO: World Health Organization

## 1 Introduction

Alcohol misuse represents a critical global public health concern. Prolonged heavy alcohol consumption can lead to alcohol‐associated liver disease (ALD), a spectrum of conditions ranging from simple hepatic steatosis to severe forms like fibrosis, cirrhosis, and alcohol‐associated hepatitis (Ramkissoon and Shah 2022). The World Health Organization (WHO) reported that in 2018, harmful alcohol use accounted for around 3 million deaths worldwide, accounting for 5.3% of all fatalities (Hernandez‐Evole et al. 2024). In China, alcohol consumption has increased over the last 30 years. In 2016, the WHO estimated that 22.7% of Chinese people aged 15 and above engaged in heavy episodic drinking (Hu et al. 2022). Research suggests that 90% of heavy alcohol consumers develop simple steatosis, with 10%–20% progressing to cirrhosis (Foncea et al. 2022; Husain et al. 2020). Given this substantial burden and its potential for severe health outcomes, ALD warrants further investigation.

The liver is primarily responsible for alcohol metabolism, processing over 95% of ingested ethanol, with only a minor fraction (2%–5%) eliminated via respiration, urine, and sweat (Kong et al. 2019). In the liver, the enzyme alcohol dehydrogenase (ADH) metabolizes ethanol into acetaldehyde, a toxic compound that can damage liver cells and other tissues. Subsequently, the enzyme aldehyde dehydrogenase (ALDH) converts acetaldehyde into acetate, which is then further decomposed into water and carbon dioxide, potentially causing cellular damage. Chronic excessive alcohol consumption leads to distinct pathological hallmarks of liver damage, primarily involving oxidative stress, apoptosis, and inflammatory injury (Park et al. 2023; Wang et al. 2016). Therefore, therapeutic agents that can ameliorate oxidative stress, inhibit apoptosis, or reduce inflammatory injury may theoretically provide protective effects against ethanol‐induced liver damage.

Shikonin (SKN, C_16_H_16_O_5_, CAS:517‐89‐5) is a natural naphthoquinone compound extracted from the roots of *Lithospermum erythrorhizon* . Current evidence suggests that it exhibits diverse pharmacological activities, including antioxidant (Lu et al. 2024), anti‐inflammatory (Liang et al. 2023), and antitumor properties (Yu et al. 2024), thereby attracting scientific interest worldwide. Our previous experimental study revealed that SKN could decrease the gene expression of pro‐inflammatory cytokines IFN‐γ, IL‐6, IL‐17A, and TNFα, while enhancing the expression of TGF‐β1, IL‐10, and indoleamine‐2, 3‐dioxygenase in skin allografts (Zeng et al. 2019). SKN pretreatment has been shown to reduce Con A‐induced acute liver injury caused by preventing apoptosis and autophagy through inhibition of the JNK signaling pathway (Liu et al. 2016). Furthermore, SKN was found to attenuate acetaminophen‐induced acute liver injury via inhibition of oxidative stress and inflammation (Guo et al. 2019), providing an invaluable reference for our research endeavors. While alcohol‐induced liver injury is more prevalent compared to drug‐induced liver injury, and despite SKN's potential for anti‐oxidative, anti‐apoptotic, and anti‐inflammatory responses, it remains unknown whether SKN ameliorates ALD.

This study investigated the role of SKN in providing hepatic anti‐oxidative, anti‐inflammatory, and anti‐apoptotic protection against alcohol‐induced liver disease in rats.

## 2 Materials and Methods

### 2.1 Reagents

SKN (purity > 98%) was obtained from MACKLIN (S888358, China). Silymarin (purity > 98%) was obtained from MACKLIN (S817884, China). Absolute ethanol (E20003, Cerusi, China) was diluted to a 52% alcohol solution using a hydrometer for precise measurement.

### 2.2 Animals

Forty‐eight male Wistar rats, each weighing between 180 and 220 g, were obtained from Guangdong Medical Laboratory Animal Center. Rats were kept in a specific pathogen‐free room, maintaining a clean environment at 22°C ± 2°C with a 12‐h light/dark cycle, and had free access to both food and water. All experiments received approval from the Institutional Animal Care and Use Committee of Guangdong Provincial Second Hospital of Traditional Chinese Medicine (approval number: IACUC049370).

### 2.3 Animal Modeling and Drug Administration

Following a 1‐week acclimation period, the 48 rats were randomly divided into six groups and given a standard chow diet: (1) Normal group: rats were given water only, (2) Model group: rats were given 52% alcohol, (3) Silymarin group: rats were given 52% alcohol + silymarin 100 mg/kg, (4) SKN (5 mg/kg): rats were given 52% alcohol + SKN 5 mg/kg, (5) SKN (10 mg/kg): rats were given 52% alcohol + SKN 10 mg/kg, (6) SKN (20 mg/kg): rats were given 52% alcohol + SKN 20 mg/kg. In addition to the normal group, the ALD model rats were administered escalating doses of alcohol via gavage. Specifically, 52% alcohol was administered 4 mL/kg from Day 0 to Day 3, 6 mL/kg from Day 4 to Day 7, and 10 mL/kg from Day 8 onwards. Drug intervention was given at the same time as modeling: After 4 h of alcohol administration, rats in the Silymarin group and SKN groups were treated with 100 mg/kg Silymarin. Besides, the SKN (5 mg/kg), SKN (10 mg/kg), and SKN (20 mg/kg) were treated with 5, 10, and 20 mg/kg SKN, respectively (Yang et al. 2020). An equal volume of distilled water was administered to both the Normal and Model groups. The duration of the modeling and administration was 6 weeks. After the final treatment, rats were anesthetized using isoflurane, after which blood was collected and serum was isolated by centrifuging the blood at 3000 rpm for 10 min. All rats were subjected to dissection, during which their livers were excised and weighed to calculate organ coefficients: liver index = (liver weight)/(body weight) × 100%. Subsequently, a portion of the liver tissue was frozen for the preparation of liver homogenate and protein extraction for Western blot analysis, while the remaining tissue was fixed in formalin for H&E staining and immunofluorescence examination.

### 2.4 Serum and Liver Homogenate Biochemical Assay

Hepatic injury was evaluated by measuring serum levels of ALT, AST, and ALP using commercially available assay kits from Nanjing Jiancheng Bioengineering Institute. The serum levels of TC, TG, GLU, CRE, BUN, and UA were measured using assay kits obtained from Nanjing Jiancheng Bioengineering Institute. The serum levels of IL‐1β (MM‐0047R2), TNF‐α (MM‐0180R1), IL‐6 (MM‐0190R1), and IL‐10 (MM‐0195R1) were determined using enzyme‐linked immunosorbent assay kits (Mei Mian Biology, China).

Liver homogenates (10%, w/v) were prepared in ice‐cold PBS using a high‐speed homogenizer. The levels of IL‐1β, IL‐6, IL‐10, and TNF‐α were quantified in liver homogenates. Besides, the levels of LPS, ROS, SOD, and MDA were quantified using the corresponding kits.

### 2.5 Histological Observation and Immunofluorescence

Liver tissue sections from each rat were processed for histological examination. Sections were stained using H&E and subsequently evaluated under a light microscope at 400× magnification (Bx51, OLYMPUS, Japan).

The liver tissue sections underwent sequential processing in xylene I and II for 12 min each, followed by immersion in absolute ethanol for 6 min, then in 95% ethanol and 85% ethanol for 6 min each. Subsequently, paraffin sections were subjected to antigen retrieval with sodium citrate (pH 6.0). The heating protocol consisted of medium heat for 8 min, followed by an 8‐min cessation of heating, and then low heat for 7 min. After being washed in PBS, the sections were treated with 3% hydrogen peroxide at room temperature. After 25 min, the sections were washed with PBS. Next, the tissue sections were uniformly covered by dropping 3% BSA into the histochemical circle and blocking for 30 min at room temperature. After slight drying, the primary antibody of Bax, diluted to the appropriate concentration, was dripped to cover the tissue. The sections were placed in a humidified chamber for overnight incubation at 4°C. After washing and drying, the corresponding secondary antibody was added dropwise to cover the tissue, and then the sample was incubated in the dark at room temperature for 50 min. Liver sections were subjected to antigen retrieval in a microwave oven using a repair box containing EDTA solution (pH 8.0), heated for 8 min on medium power, followed by 8 min on low power. After brief drying, the sections were treated with DAPI staining solution and left to incubate in the dark at room temperature. Ten minutes later, the sections were washed with PBS, then gently dried and sealed using an anti‐quenching agent for fluorescence. The images were captured with a fluorescence microscope at 200× magnification (Ti2‐U, Nikon Instruments, Japan).

### 2.6 Western Blotting Analysis

RIPA lysis buffer was used to extract protein from the liver tissues of each group. The proteins underwent electrophoresis using 10% SDS‐PAGE gels and were then transferred to PVDF membranes via electroblotting. The membranes were incubated at 4°C overnight with specific primary antibodies from Proteintech China targeting TLR4 (#19811‐1‐AP, 1:1000), MYD88 (#23230‐1‐AP, 1:5000), NF‐κB (P65) (#10745‐1‐AP, 1:1000), HO‐1 (#10701‐1‐AP, 1:1000), Keap‐1 (#30041‐1‐AP, 1:5000), Nrf2 (#16396‐1‐AP, 1:2000), Bax (#50599‐2‐Ig, 1:2000), Bcl‐2 (#26593‐1‐AP, 1:1000), and Caspase‐3 (#25128‐1‐AP, 1:1000 dilution). Subsequently, the membranes were washed and incubated with a secondary antibody at room temperature for 2 h. GAPDH (D16H11, 1:8000, Cell Signaling Technology, USA) served as a loading control. The blots were finally visualized using an ECL detection system.

### 2.7 Statistical Analysis

Data analysis was conducted using SPSS version 19.0. One‐way ANOVA was used to compare normally distributed variables among multiple groups, followed by Fisher's least significant difference (LSD) post hoc test for pairwise comparisons. The results were expressed as the mean ± standard error of the mean (SEM). The symbol “*n*” represents the biological replicates. A *p* value < 0.05 was statistically significant.

## 3 Results

### 3.1 SKN Ameliorated Alcohol‐Induced Liver Injury in Rats

**FIGURE 1:**
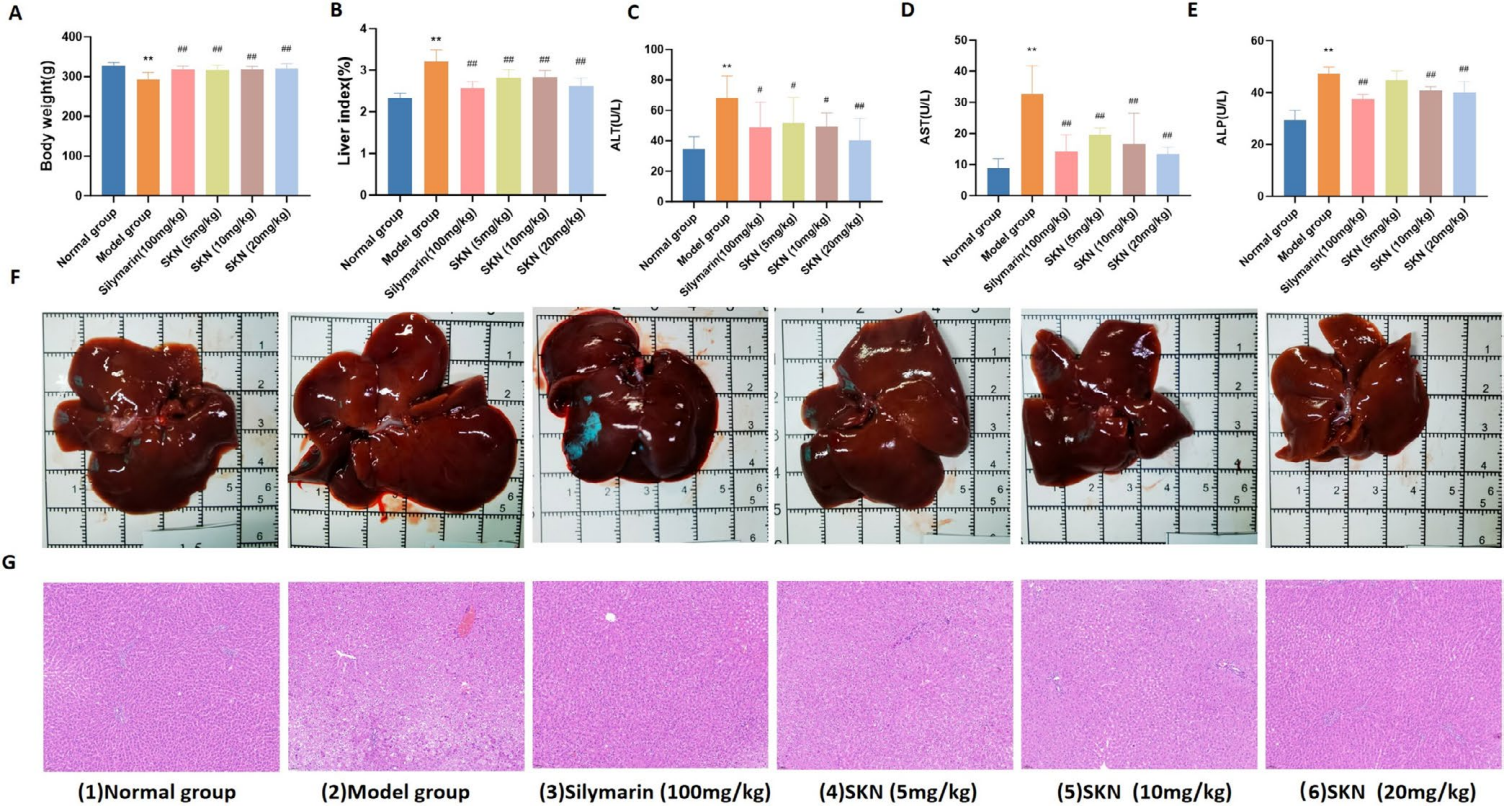
SKN improved liver damage caused by alcohol in rats. (A, B) Body weight and liver indices ($\bar{x}$ ± *s*, *n* = 8). **p < 0.01 versus Normal group, ^##^ *p* < 0.01 versus Model group. (C–E) Serum levels of AST, ALT, and ALP were quantified using corresponding kits ($\bar{x}$ ± *s*, *n* = 8). **p < 0.01 versus Normal group, ^#^ *p* < 0.05 versus Model group, ^##^ *p* < 0.01 versus Model group. (F) Evaluation of liver status. (G) Liver section analyzed pathologically (5 μm thick, H&E staining, 200× magnification).

After alcohol administration, rats in all groups except the Normal group exhibited signs of excitement, characterized by unsteady gait and rapid breathing. As depicted in Figure 1A,B, body weight was reduced and liver index was elevated in the Model group relative to the normal group (*p* < 0.01). Treatment with Silymarin (100 mg/kg) and SKN at doses of 20, 10, and 5 mg/kg mitigated the impact of ALD on these parameters.

As shown in Figure 1C–E, alcohol intake in the Model group led to a significant increase in serum AST, ALT, and ALP compared to the control group (*p* < 0.01). Treatment with Silymarin (100 mg/kg) and SKN at doses of 5, 10, and 20 mg/kg demonstrated a dose‐dependent reduction in AST and ALT levels compared to the Model group (*p* < 0.01). Besides, SKN at 10 and 20 mg/kg significantly decreased ALP levels (*p* < 0.01).

The morphological characteristics of the rat liver were next examined, as shown in Figure 1F. The livers in the Normal group exhibited typical characteristics: normal shape, moderate size, reddish‐brown color, uniform texture, and sharp edges. In contrast, livers in the model group showed significant pathological changes, including increased volume, pronounced surface congestion with a dark red hue, and visible yellow particles on the surface, along with blunted edges. With increasing doses of SKN, liver injury in the 5, 10, and 20 mg/kg dose groups was alleviated to varying degrees. These livers appeared reddish‐brown with a more uniform texture and sharp edges, characterized by reduced volume increase and lessened surface congestion. In the silymarin group, the liver volume was only slightly enlarged, with a few yellow particles observed on the surface and slightly blunted edges.

H&E staining (Figure 1G) demonstrated that hepatocytes in the Normal group maintained their structural integrity, exhibiting uniform size, and a characteristic radial arrangement around the central vein of the hepatic lobule. The cytoplasm was evenly stained red, featuring large, rounded nuclei, and no lesions were detected. The Model group displayed notable liver tissue disorganization, characterized by the loss of hepatic cords and enlarged hepatocytes. There was a significant reduction or absence of nuclei, irregular cell shapes, and numerous fat vacuoles, accompanied by inflammatory infiltration. Administration of silymarin and SKN at doses of 5, 10, and 20 mg/kg significantly improved hepatic fat vacuole patterns and reduced inflammatory cell infiltration compared to the Model group.

### 3.2 SKN Mitigated Metabolic Disturbances in ALD in Rats

**FIGURE 2:**
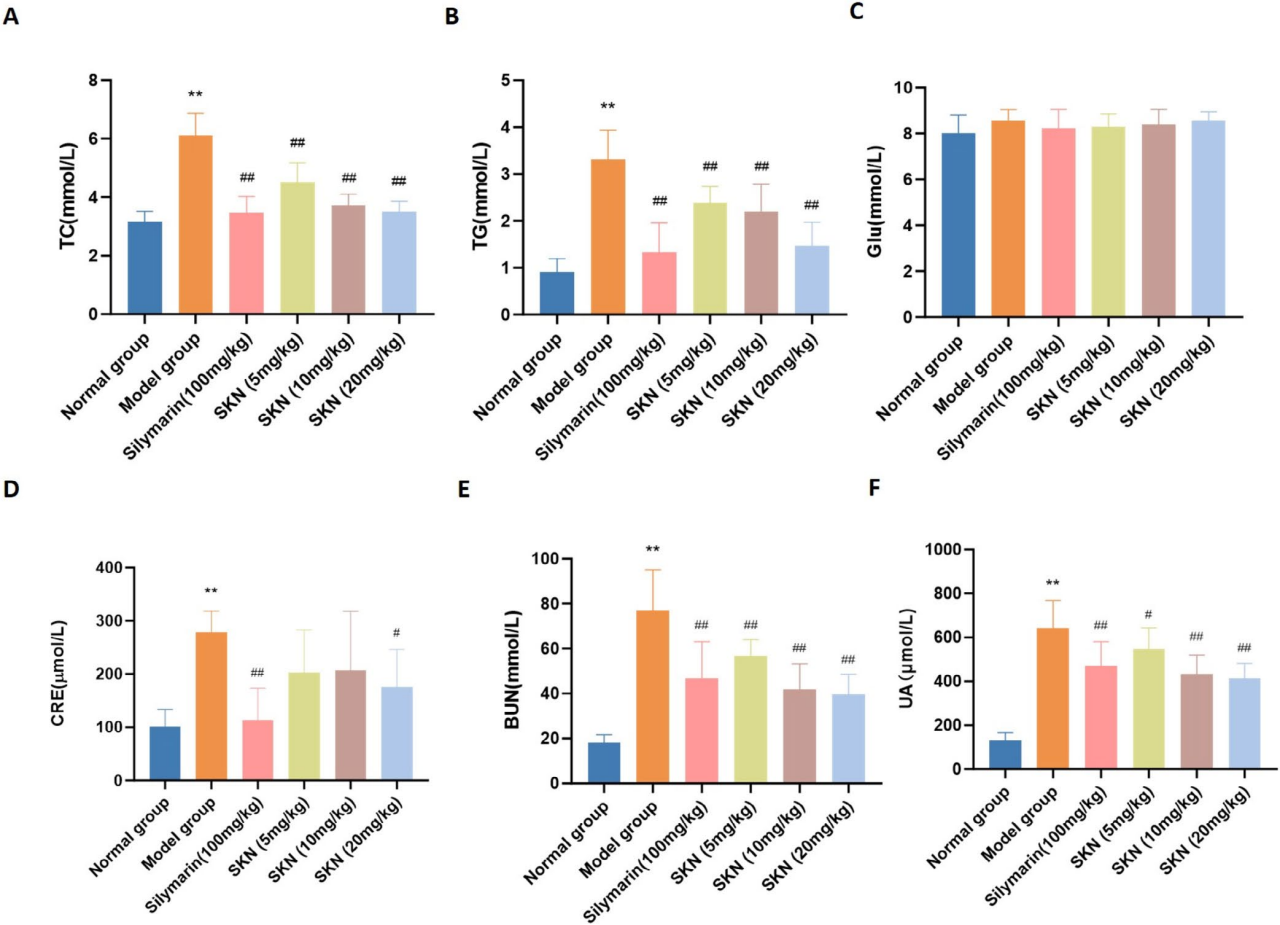
SKN mitigated metabolic disturbances in ALD rat models. (A–C) Serum TC, TG, and Glu were assessed using kits ($\bar{x}$ ± *s*, *n* = 8). **p < 0.01 versus Normal group, ^##^ *p* < 0.01 versus Model group. (D–F) Serum levels of CRE, BUN, and UA were measured using corresponding kits ($\bar{x}$ ± *s*, *n* = 8). **p < 0.01 versus Normal group, ^#^ *p* < 0.05 versus Model group, ^##^ *p* < 0.01 versus Model group.

To assess the impact of SKN on abnormalities in glucose and lipid metabolism associated with alcohol‐induced liver injury, serum biochemical parameters were analyzed. As shown in Figure 2A–C, serum TG and TC experienced a significant rise in the Model group compared with those in the Normal group (*p* < 0.01). Pretreatment with Silymarin (100 mg/kg) and SKN at doses of 5, 10, and 20 mg/kg substantially inhibited the alcohol‐induced elevation in both serum TG and TC levels (*p* < 0.01). Concurrently, there were no significant differences in blood glucose among the experimental groups (*p* > 0.05).

We further evaluated the renal function indicators of the rats, with the results presented as Figure 2D–F. The levels of renal function markers, including CER, BUN, and UA, were significantly higher in the Model group. The CER levels significantly decreased in the Silymarin (100 mg/kg) and SKN (20 mg/kg) groups compared to the Model group (*p* < 0.05 and *p* < 0.01, respectively). In the SKN groups, BUN and UA levels decreased in a concentration‐dependent manner (*p* < 0.05 and *p* < 0.01, respectively).

### 3.3 SKN Treatment Decreased Oxidative Stress in ALD in Rats

**FIGURE 3:**
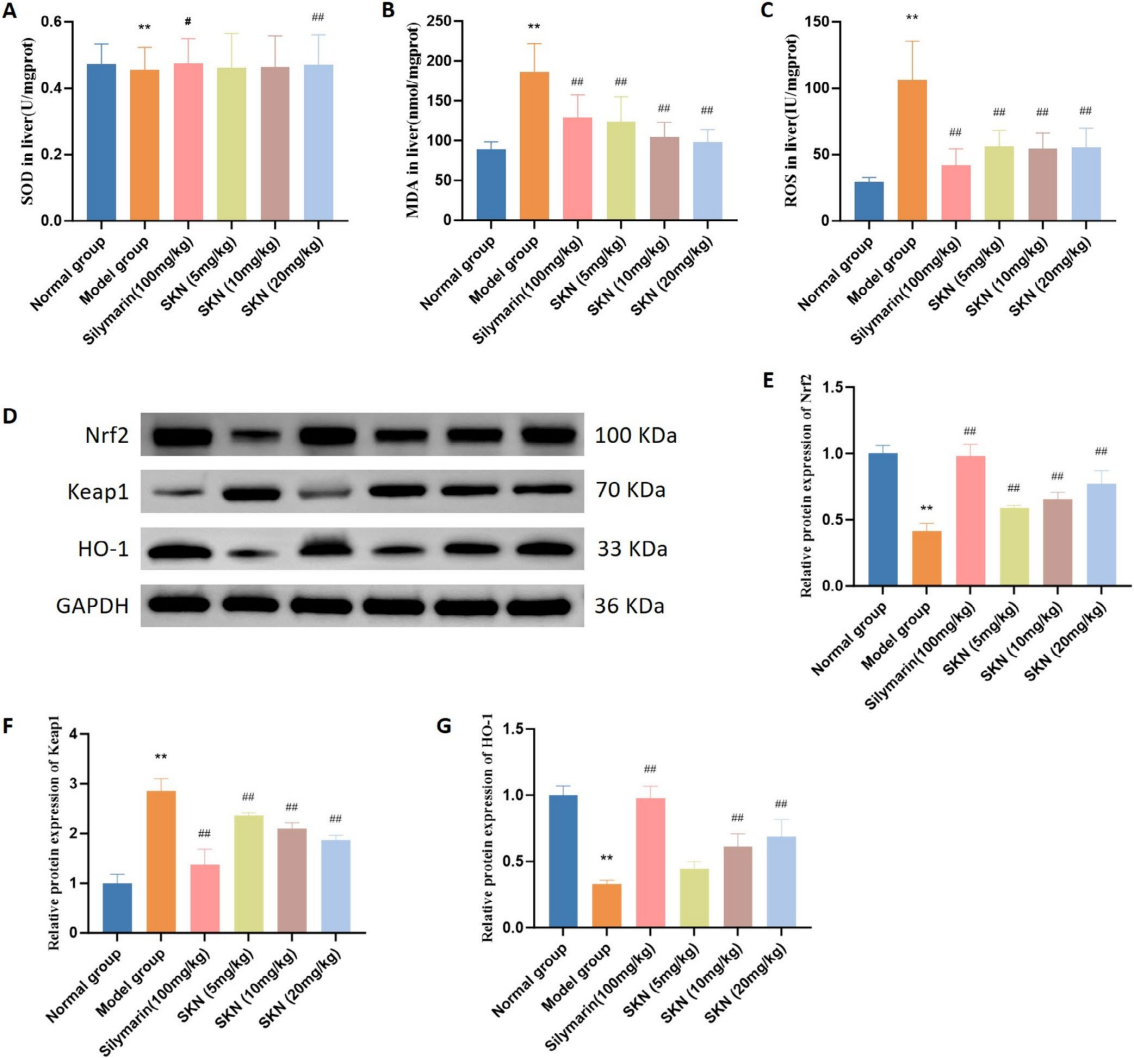
SKN treatment decreased oxidative stress in ALD rat models. (A–C) Levels of SOD, MDA, and ROS in the liver were measured using corresponding kits ($\bar{x}$ ± *s*, *n* = 8). **p < 0.01 versus Normal group, ^#^ *p* < 0.05 versus Model group, ^##^ *p* < 0.01 versus Model group. (D) Protein expression of Nrf2/Keap1/HO‐1 in the liver was assessed by Western blot analysis. (E–G) Relative protein expression of Nrf2, Keap1, and HO‐1 was normalized to GAPDH expression ($\bar{x}$ ± *s*, *n* = 3). **p < 0.01 versus Normal group, ^##^ *p* < 0.01 versus Model group.

Alcohol exposure significantly induces oxidative stress and dysfunction. As shown in Figure 3A–C, a significant reduction in SOD activity and an increase in MDA, a lipid peroxidation marker, were observed in the Model group following alcohol exposure. However, compared with the Model group, Silymarin (100 mg/kg) and SKN (20 mg/kg) significantly increased the levels of SOD (*p* < 0.05 and *p* < 0.01, respectively). Besides, Silymarin (100 mg/kg) and all doses of SKN (5, 10, and 20 mg/kg) decreased MDA levels in the liver (all *p* < 0.01). In the Model group, ROS activity exhibited a significant increase. Compared to the Model group, Silymarin (100 mg/kg) and SKN (5, 10, and 20 mg/kg) significantly diminished ROS activity in the liver (all *p* < 0.01).

Western Blot was next conducted to evaluate the protein expression of Keap1, Nrf2, and HO‐1 in the liver, further validating the capacity of SKN to mitigate oxidative stress. As shown in Figure 3D–G, alcohol administration significantly inhibited the protein expression of Nrf2 and HO‐1, while upregulating Keap1 in liver tissues (*p* < 0.01). After treatment, Silymarin (100 mg/kg) and all tested doses of SKN (5, 10, and 20 mg/kg) significantly reduced Keap1 protein expression while enhancing Nrf2 protein expression (all *p* < 0.01). Besides, Silymarin (100 mg/kg) and SKN at 10 and 20 mg/kg significantly elevated HO‐1 protein expression (*p* < 0.01), indicating that SKN activated the Keap1/Nrf2/HO‐1 signaling pathway.

### 3.4 SKN Mitigated the Inflammatory Response in ALD Rat Models

**FIGURE 4:**
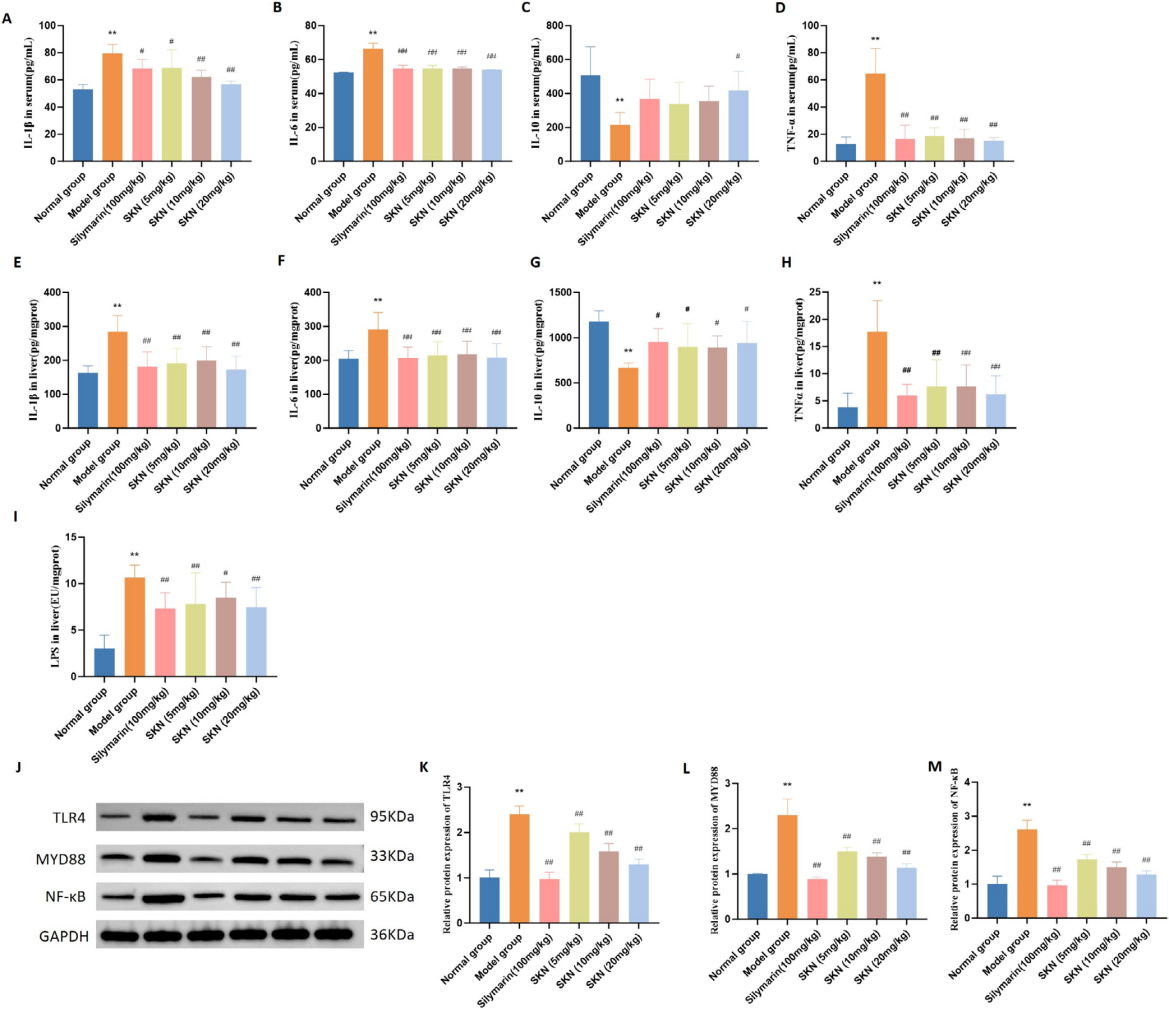
SKN treatment decreased the inflammatory response in ALD rat models. (A–D) Measurement of serum concentrations of IL‐1β, IL‐6, IL‐10, and TNF‐α ($\bar{x}$ ± *s*, *n* = 8). **p < 0.01 versus Normal group, ^#^ *p* < 0.05 versus Model group, ^##^ *p* < 0.01 versus Model group. (E–I) Measurement of IL‐1β, IL‐6, IL‐10, TNF‐α, and LPS concentrations in liver ($\bar{x}$ ± *s*, *n* = 8). **p < 0.01 versus Normal group, ^#^ *p* < 0.05 versus Model group, ^##^ *p* < 0.01 versus Model group. (J) Protein expression levels of TLR4, MYD88, and NF‐κB in liver were assessed using Western blot analysis. (K–M) Relative protein expression of TLR4, MYD88, and NF‐κB was normalized to GAPDH expression ($\bar{x}$ ± *s*, *n* = 3). **p < 0.01 versus Normal group, ^##^ *p* < 0.01 versus Model group.

Alcoholic liver toxicity development is related to activation of the innate immune response, characterized by a rise in pro‐inflammatory cytokines such as IL‐6, TNF‐α, and IL‐1β. Furthermore, IL‐10, a well‐established anti‐inflammatory cytokine, plays a critical role in modulating this response. As shown in Figure 4A–D, serum levels of IL‐1β, IL‐6, and TNF‐α were significantly elevated in the Model group (*p* < 0.01). Pretreatment with Silymarin (100 mg/kg) and SKN (5, 10, and 20 mg/kg) significantly reduced serum levels of pro‐inflammatory cytokines. The Model group exhibited significantly lower serum IL‐10 levels compared to the Normal group (*p* < 0.01). Serum IL‐10 levels were markedly raised in the SKN (20 mg/kg) group compared with the Model group (*p* < 0.05). Moreover, similar results were documented for inflammatory cytokines in the liver. As shown in Figure 4E–I, Silymarin (100 mg/kg) and SKN at doses of 5, 10, and 20 mg/kg significantly reduced IL‐6, TNF‐α, IL‐1β, and LPS levels in liver tissue (*p* < 0.05 and *p* < 0.01), while simultaneously elevating IL‐10 levels (*p* < 0.05). As shown in Figure 4J–M, Silymarin (100 mg/kg), SKN (5 mg/kg), SKN (10 mg/kg), and SKN (20 mg/kg) significantly reduced the expression of TLR4, MYD88, and NF‐κB proteins to nearly normal levels (all *p* < 0.01).

### 3.5 SKN Inhibited Apoptosis in ALD Rat Models

**FIGURE 5:**
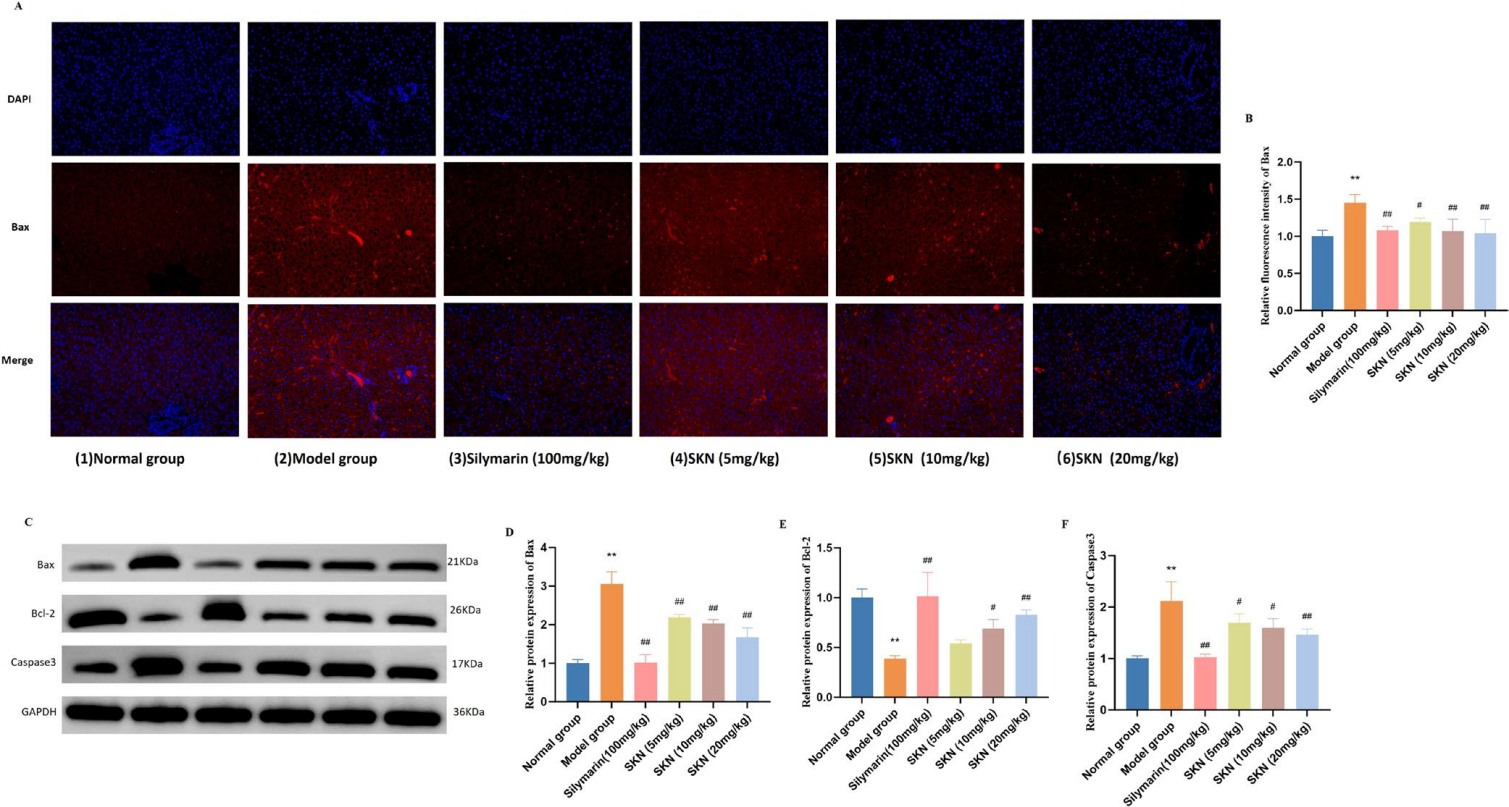
SKN inhibited apoptosis in ALD rat models. (A) Immunofluorescence staining of Bax. (B) Relative fluorescence intensity of Bax ($\bar{x}$ ± *s*, *n* = 3). **p < 0.01 versus Normal group, ^#^ *p* < 0.05 versus Model group. (C) Western blotting was used to assess the liver protein expression levels of Bax, Bcl‐2, and Caspase3. (D–F) Relative protein expression of Bax, Bcl‐2, and Caspase3 was normalized to GAPDH expression ($\bar{x}$ ± *s*, *n* = 3). **p < 0.01 versus Normal group, ^#^ *p* < 0.05 versus Model group, ^##^ *p* < 0.01 versus Model group.

Immunofluorescence analysis was next used to assess the staining intensity of Bax in the liver of ALD rat models (Figure 5A,B). Compared with the Normal group, Bax expression was significantly upregulated after alcohol administration. Nevertheless, treatment with Silymarin (100 mg/kg) and all doses of SKN (5, 10, and 20 mg/kg) significantly reduced Bax expression (*p* < 0.05 and *p* < 0.01, respectively). To further confirm the ability of SKN to inhibit apoptosis, protein expression levels of Bax, Bcl‐2, and Caspase3 in the liver were analyzed through western blotting. As shown in Figure 5C–F, treatment with Silymarin (100 mg/kg) and SKN (5, 10, and 20 mg/kg) significantly decreased Bax and Caspase3 protein expression (*p* < 0.05 and *p* < 0.01), while Silymarin (100 mg/kg) and SKN (10 and 20 mg/kg) substantially upregulated Bcl‐2 protein expression levels (*p* < 0.05 and *p* < 0.01). These results collectively indicate that SKN treatment effectively inhibited the apoptosis of hepatic cells in rat models of alcohol‐associated liver disease.

## 4 Discussion

ALD encompasses various liver conditions caused by long‐term excessive alcohol consumption, such as alcoholic fatty liver disease, alcoholic hepatitis, liver fibrosis, and potentially hepatocellular carcinoma. Owing to the increasing prevalence of excessive alcohol consumption, the population affected by ALD is progressively expanding. Currently, complete abstinence from alcohol represents the most effective intervention for ALD. It is widely thought that the advent of pharmacotherapies that can mitigate the hepatotoxic effects of alcohol is vital for managing this condition. Importantly, the developmental strategies for drugs targeting the treatment of ALD primarily emphasize accelerating ethanol metabolism, mitigating inflammatory damage, preventing peroxidation, and providing symptomatic treatment to alleviate symptoms. Numerous studies have demonstrated that acute alcoholic liver injury is characterized by a mutually reinforcing cascade involving oxidative stress, inflammation, and apoptosis (Diaz et al. 2023). Specifically, oxidative stress triggers inflammatory responses, which, in turn, exacerbate oxidative damage. The synergistic effect of oxidative stress and inflammation prompts hepatocyte apoptosis. Moreover, the release of damage‐associated molecular patterns (DAMPs) from apoptotic cells further enhances both inflammation and oxidative stress, thereby creating a self‐perpetuating cycle. This study sought to elucidate the mechanism of action and evaluate the therapeutic efficacy of SKN in modulating the aforementioned signaling pathways involved in acute alcoholic liver injury. SKN, the primary bioactive compound derived from Zicao, has traditionally been used to treat various infectious and inflammatory diseases (Li et al. 2018). Our previous studies have demonstrated that SKN exhibits significant anti‐inflammatory, antioxidant, and immunomodulatory properties, prompting further investigation of the effects of SKN on ALD. Our current research indicated that SKN treatment offers a protective effect against alcohol hepatotoxicity by inhibiting oxidative stress and the inflammatory response, suggesting its potential as a viable therapeutic agent against ALD.

The liver is the primary organ responsible for alcohol metabolism, making it highly susceptible to alcohol‐induced damage. Meanwhile, clinical studies have shown that chronic alcoholics commonly experience weight loss due to calorie displacement from ethanol and inadequate intake of protein and vitamins (Gray et al. 2019; Kimball and Lang 2018). Therefore, in animal models, body weight, liver index, and liver function indicators serve as reliable measures of hepatic injury. Liver cell damage compromises transport function and membrane permeability, resulting in the release of enzymes such as ALT, AST, and ALP into the bloodstream. Elevated serum activity of these enzymes directly correlates with the extent of liver damage. Serum levels of ALT, AST, and ALP are crucial and sensitive biochemical markers of liver function, offering an early indication of ALD. This study found that SKN treatment significantly increased body weight and reduced liver index and serum ALT, AST, and ALP levels in rats with alcoholic liver injury. Histopathological analysis indicated that SKN treatment markedly alleviated liver hemorrhage, fat accumulation, necroinflammation, hepatocyte swelling, and necrosis in rats with alcoholic liver injury, nearly restoring normal liver histology. These findings provide compelling evidence that SKN ameliorates ALD progression in rats by enhancing intestinal nutrient absorption and suppressing hepatic damage, as evidenced by reduced serum ALT, AST, and ALP activity, attenuated histopathological inflammatory cell infiltration, and decreased hepatocellular necrosis, thereby confirming its hepatoprotective properties.

It is now understood that alcohol significantly affects hepatic lipid metabolism, influencing various aspects of lipid flux and leading to lipid accumulation. Chronic alcohol consumption directly contributes to steatosis or fatty liver by impeding hepatic fat outflow, thereby elevating blood concentrations of TC, TG, and other fats, which accumulate in liver tissue (Alpert and Hart 2016). At the same time, studies have shown that the detrimental effects of heavy alcohol consumption on kidney structure can persist, leading to metabolic disorders of CRE, BUN, and UA after long‐term withdraw (Leal et al. 2017). In this study, following alcohol administration, serum levels of TC, TG, CRE, BUN, and UA in rats were significantly elevated compared to the normal group. However, SKN intervention markedly attenuated these increases: TC and TG levels were reduced, indicating improved lipid metabolism and mitigation of hepatic steatosis. Concurrently, CRE, BUN, and UA levels declined, suggesting alleviation of alcohol‐induced renal impairment. Interestingly, blood glucose levels remained stable in both normal and alcohol‐treated groups, warranting further investigation into the underlying mechanisms.

Oxidative stress, induced by alcohol in the liver, is a key mechanism underlying alcoholic liver injury. Alcohol induces the production of a significant amount of ROS, overwhelming the antioxidant defense system capacity of the liver, leading to hepatic cell damage, and promoting the progression of alcoholic liver disease (Tan et al. 2020). During oxidative stress, SOD serves as a crucial free radical scavenger within the body, protecting it from peroxidation‐induced damage. Concurrently, MDA, a cytotoxic byproduct of lipid peroxidation caused by free radicals, exhibits cytotoxic properties, which can indirectly reflect the degree of tissue damage. Our study demonstrated that SKN administration resulted in a reduction of ROS and MDA levels, alongside an increase in SOD content in the livers of rats with alcohol‐induced liver injury. These findings suggest that SKN may mitigate oxidative stress damage in the liver and exhibit antioxidant properties. Accordingly, the expression of the Keap1/Nrf2/HO‐1 pathway was assessed to clarify SKN's antioxidant mechanism in alcohol‐induced liver injury. The Nrf2 transcription factor is a crucial regulator of cellular defense against oxidative stress, triggering the antioxidant response and enhancing the production of antioxidant proteins and enzymes. In normal cells, Nrf2 levels are maintained at low concentrations in the cytoplasm through dimerization and binding to Keap1, facilitating its polyubiquitination and subsequent proteasomal degradation (Tu et al. 2019). Under stress conditions, elevated endogenous or exogenous ROS levels cause Nrf2 to dissociate from Keap1 and translocate to the nucleus, where it regulates and enhances HO‐1 activity (Ma 2013). HO‐1 is an essential antioxidant enzyme that safeguards cells by producing carbon monoxide and breaking down heme to produce bilirubin, neutralizing harmful oxygen free radicals and reducing oxidative stress damage (Costa and Correa 2022). Western blot analysis revealed that SKN significantly upregulated nuclear Nrf2 protein expression while downregulating Keap1 levels, thereby disrupting the Keap1–Nrf2 interaction and activating the antioxidant response element‐driven transcription of HO‐1. This coordinated modulation of the Nrf2/Keap1/HO‐1 axis effectively ameliorated alcohol‐induced oxidative stress markers, including reduced hepatic MDA accumulation and restored SOD. Mechanistically, these findings suggest SKN alleviates hepatocyte injury by enhancing endogenous antioxidant defense systems through the Nrf2/Keap1/HO‐1 axis.

Alcoholic liver injury is characterized by sustained hepatocyte damage and persistent inflammation, which constitutes a complex reaction to stress and death in hepatocytes. Chronic excessive alcohol intake increases intestinal permeability, enabling the translocation of LPS via the portal vein to the liver. This systemic release subsequently activates inflammatory cells, such as macrophages, which then secrete IL‐1β, IL‐6, and TNF‐α (Zhang and Gao 2021). Clinical studies have identified elevated levels of cytokines TNF‐α, IL‐1, and IL‐6 in patients with alcoholic liver disease (Khoruts et al. 1991). Conversely, IL‐10, an anti‐inflammatory cytokine, exhibited an inverse correlation with psychiatric symptoms in patients with excessive alcohol intake (Bjorkhaug et al. 2020). Our results suggested that SKN intervention could significantly reduce the levels of TNF‐α, IL‐1β, and IL‐6 while increasing IL‐10 levels in both serum and liver compared to the model group, indicating notable anti‐inflammatory activity. According to the results mentioned, the TLR4/MyD88/NF‐κB signaling pathway was selected to explore SKN's anti‐inflammatory mechanism. A study reported that chronic alcohol intake can disrupt gut microbiota balance and tight junction protein expression, resulting in elevated intestinal permeability and LPS levels in vivo (Jung et al. 2021). TLR4 is an exogenous receptor specifically recognizing LPS. Following significant alcohol consumption, TLR4 is activated by LPS, subsequently activating the adaptor protein MyD88. Activated MyD88 then triggers downstream NF‐κB, facilitating the release of inflammatory factors (Pandey 2012). In summary, alcohol consumption elevates LPS levels in vivo, which subsequently activates NF‐κB and promotes the secretion of pro‐inflammatory cytokines by upregulating the TLR4/MyD88 signaling pathway. In this study, the LPS content was markedly elevated in the rat models of alcoholic liver disease. Furthermore, the protein expression levels of TLR4, MyD88, and NF‐κB were significantly upregulated. These results aligned with prior research findings (Liu et al. 2023). Besides, the SKN groups exhibited reduced serum LPS levels and decreased hepatic protein expression of TLR4, MyD88, and NF‐κB. This study demonstrated that SKN could ameliorate alcohol‐induced hepatic inflammation by suppressing TLR4‐mediated MyD88‐dependent signaling. Mechanistically, SKN inhibited TLR4 homodimerization and subsequent MyD88 recruitment, leading to impaired nuclear translocation of NF‐κB. Consequently, this downregulation of the TLR4/MyD88/NF‐κB axis reduced pro‐inflammatory cytokine production (IL‐1β, IL‐6, and TNF‐α) and increased IL‐10, the anti‐inflammatory factor secretion, thereby mitigating liver inflammatory injury and hepatocyte apoptosis.

As mentioned above, chronic alcohol exposure induces a mitochondrial redox imbalance through electron transport chain uncoupling and the inhibition of complexes I/III, resulting in the overproduction of ROS that surpasses endogenous antioxidant defenses. This oxidative overload triggers lipid peroxidation and oxidative damage to macromolecules. ROS‐mediated disruption of hepatocellular membrane integrity activates redox‐sensitive NF‐κB and the subsequent pro‐inflammatory cytokine cascade. These cytokines, especially TNF‐α, further amplify mitochondrial ROS, establishing a self‐amplifying oxidative‐inflammatory loop. Concurrently, ROS promotes the opening of the mitochondrial permeability transition pore by oxidizing adenine nucleotide translocase, thereby facilitating the release of cytochrome c and apoptotic body formation. This activates executioner caspase‐3, while Bcl‐2 family dysregulation and oligomerization of the proapoptotic protein Bax induce mitochondrial outer membrane permeabilization, ultimately driving caspase‐dependent apoptosis. Research indicates that chronic alcohol consumption can lead to abnormal apoptosis of hepatocytes in vivo, with the level of apoptosis positively connected with the severity of liver damage due to alcohol (Zwolak et al. 2016). Besides, current evidence suggests that the initiation and progression of hepatocyte apoptosis involve numerous genes, notably the Bcl‐2 and Bax genes. Distinct protein dimers exert unique influences on apoptosis, underscoring the vital role of the Bcl‐2 protein family in managing cell death signaling pathways and serving as a crucial domain for apoptosis key regulators (Warren et al. 2019). It has been reported that the protein expression levels of Bcl‐2 and Bax are generally stable. When Bax is overexpressed, it enhances cell responsiveness to death signals and initiates apoptosis. Conversely, high expression of Bcl‐2 leads to Bax/Bax dimer dissociation and the construction of stable Bcl‐2/Bax heterodimers, which counteract apoptosis and promote cell survival (Kale et al. 2018). Functionally, Bax is regarded as a proapoptotic protein that primarily enhances the mitochondrial membrane's permeability, aiding in the release of cytochrome c or other intracellular components associated with apoptosis into the cytoplasm, thereby initiating the apoptotic cascade. In contrast, Bcl‐2 acts as an anti‐apoptotic protein, protecting cells from apoptosis by inhibiting proapoptotic factors, such as Bax. Bax, conversely, promotes apoptosis by increasing the permeability of the mitochondrial membrane, thereby aiding in the release of cytochrome c and other intracellular components. The members of the Bcl‐2 family can interact with non‐Bcl‐2 family proteins and exert functions beyond mutual activation or inhibition. For instance, Bax regulates caspase activation, especially caspase‐3 (Zhang et al. 1998), which facilitates apoptosis by cleaving and subsequently inactivating structural and regulatory proteins in both the nucleus and cytoplasm, which has been established as the principal executor of apoptosis within the caspase cascade (Eskandari and Eaves 2022). In this study, immunofluorescence analysis revealed that SKN significantly decreased the expression levels of Bax in the liver. At the same time, western blotting results indicated that SKN could reduce Bax and caspase‐3 levels while increasing Bcl‐2 expression. Besides, SKN could attenuate alcohol‐induced hepatocyte apoptosis by restoring mitochondrial apoptotic homeostasis. Mechanistically, SKN suppressed the mitochondrial translocation of pro‐apoptotic Bax while upregulating Bcl‐2 expression. This intervention effectively blocked Bax oligomerization on the mitochondrial outer membrane, thereby preventing mitochondrial permeability transition pore opening and subsequent cytochrome c release. At the same time, SKN inhibited caspase‐3 activation, enhancing Bcl‐2 stability. These multi‐targeted modulations collectively validate SKN's anti‐apoptotic potency through the regulation of the intrinsic mitochondrial pathway.

**FIGURE 6:**
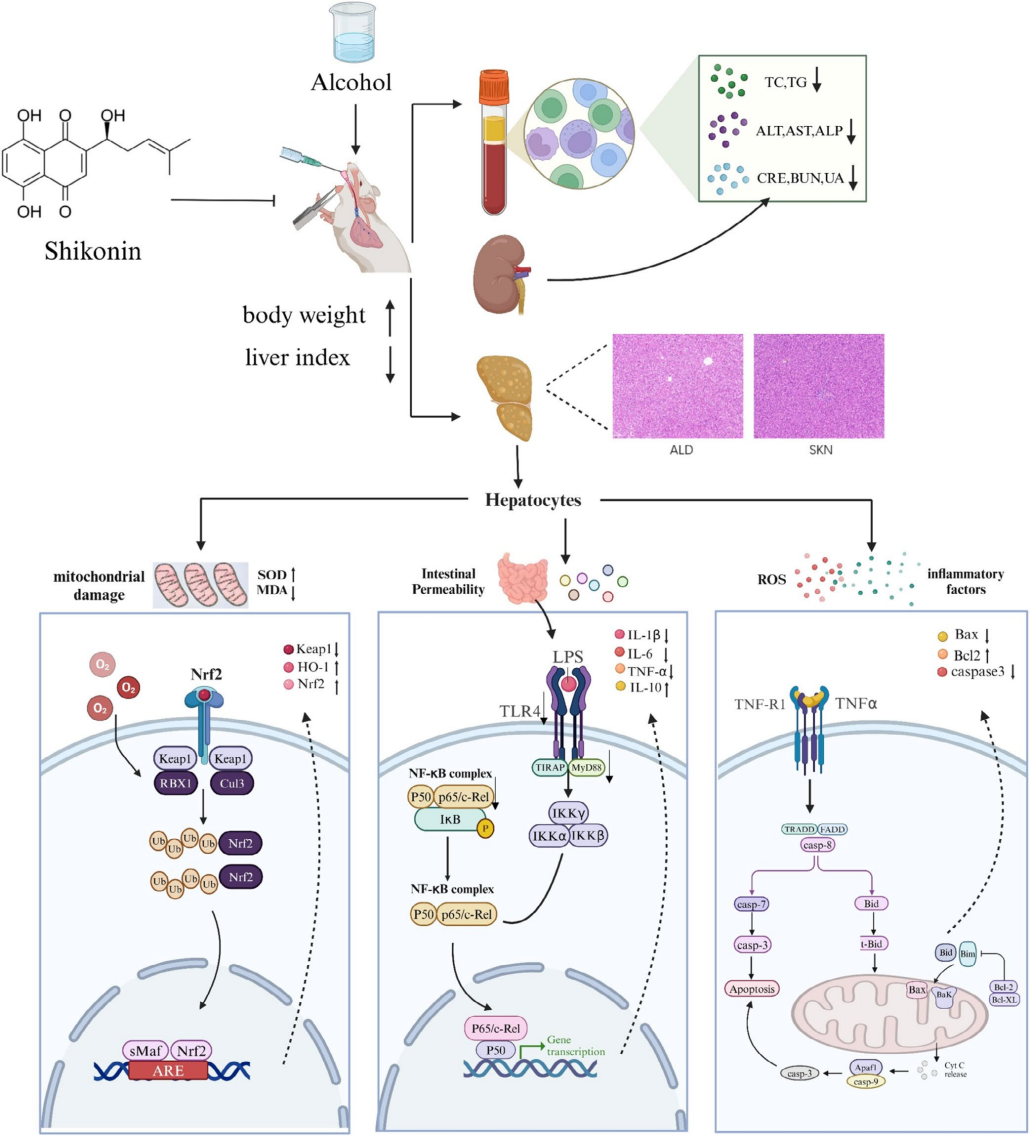
Potential mechanisms by which SKN alleviates ALD. ↑ indicates increase, ↓ indicates inhibition.

This study also revealed that SKN pretreatment protects against alcohol‐related liver injury and examined the underlying mechanism. Our results indicated that SKN modulated the Keap1/Nrf2/HO‐1 pathway to activate the antioxidant defense system, thereby reducing oxidative stress. Moreover, it mitigated inflammation by downregulating the TLR4/MyD88/NF‐κB signaling pathway. Furthermore, SKN modulated the Bax/Bcl‐2/Caspase‐3 signaling pathway to prevent hepatocyte apoptosis. Overall, our research indicates that SKN could be a promising therapeutic agent for ALD (Figure 6).

This research highlights several natural products for ALD treatment, including silymarin, curcumin, resveratrol, and others, each with well‐defined targets. Silymarin, a well‐established hepatoprotective agent, primarily acts via antioxidant (Nrf2 pathway) and antifibrotic (TGF‐β inhibition) mechanisms. However, its utility is limited by low oral bioavailability (< 10%) (Zhu et al. 2013). Curcumin strongly inhibits the NF‐κB inflammatory pathway with high safety, yet suffers from poor bioavailability (approximately 1% orally) (Yang et al. 2007). While resveratrol can reportedly modulate the SIRT1 metabolic pathway (Lagouge et al. 2006), extensive intestinal/hepatic metabolism (glucuronidation/sulfation) limits its bioavailability to 0.5%–2% (Mattarei et al. 2014). SKN demonstrates unique advantages by targeting three pathways: (1) Reducing gut‐liver LPS leakage (potential gut barrier restoration); (2) Inhibiting the TLR4/MyD88‐NF‐κB axis (similar to curcumin); (3) Enhancement of antioxidant enzyme activity (similar to resveratrol). This multi‐target synergy, combined with higher bioavailability, positions SKN as a promising candidate. At the same time, the potential coadministration of SKN with other natural medications necessitates careful consideration due to the risk of unanticipated pharmacodynamic interactions. For instance, the concurrent use of SKN and silymarin could lead to overactivation of Nrf2, potentially inducing excessive antioxidant stress, and risking redox imbalance (Lian et al. 2023). In contrast, SKN exhibits synergistic effects in antioxidant and anti‐inflammatory actions when combined with curcumin (Lu et al. 2015). Furthermore, coadministration of SKN and resveratrol significantly diminishes inflammatory factors while enhancing oxidative stress defense in a synergistic manner. This is achieved via dual inhibition of NF‐κB via SIRT1 activation and TLR4 blockade, alongside a cross‐regulatory mechanism between Nrf2 and SIRT1 (Tao et al. 2016; Wang et al. 2024). The abovementioned findings regarding the combination of SKN with other natural products warrant validation through subsequent experimental investigations. In addition, SKN, like many other natural products, presents the challenge of low bioavailability. Future application development should focus on exploring and innovating new dosage forms to effectively enhance their bioavailability.

Our prior study found that administering SKN at 40 mg/kg for 28 days did not significantly affect liver (ALT and AST) or kidney function (CER and BUN) (as shown in Figure S1). Importantly, this study revealed that SKN exhibited no dose‐dependent adverse effects across the tested dosage spectrum (5, 10, and 20 mg/kg). Notably, high‐dose SKN (20 mg/kg) significantly ameliorated alcohol‐induced multi‐organ dysfunction, as evidenced by hepatic protection (ALT and AST) and renal protection (CRE and BUN). Histopathological analysis further confirmed the absence of hepatorenal toxicity, with no marked necrosis, inflammatory infiltration, or fibrosis in SKN‐treated groups. Therefore, SKN demonstrated a favorable safety profile at doses of 5, 10, and 20 mg/kg under experimental conditions.

Finally, this research has several limitations that should be acknowledged, including the multifaceted nature of alcohol‐induced tissue effects and the complex interactions among various organs and tissues. Indeed, the complex pathogenesis of ALD involves extensive crosstalk between hepatic, intestinal, and systemic immune compartments, which were not comprehensively modeled in our liver‐focused experimental design. Future investigations should prioritize SKN's effects on gut barrier restoration to comprehensively evaluate its potential as an anti‐ALD agent. Addressing the aforementioned limitations systematically in future research will be crucial to comprehensively validate SKN as a promising therapeutic strategy for ALD.

## Author Contributions

**Haining Gan:** conceptualization (equal), data curation (lead), formal analysis (lead), funding acquisition (lead), writing – original draft (lead). **Miaoling Gong:** conceptualization (equal), data curation (equal), formal analysis (equal). **Lingzhi Wang:** conceptualization (equal), data curation (equal), investigation (equal). **Xuejun Huang:** conceptualization (equal), project administration (lead). **Yuxing Chen:** conceptualization (equal), supervision (equal). **Zixuan Hu:** conceptualization (equal), investigation (equal). **Xuewen Li:** conceptualization (equal), formal analysis (equal), funding acquisition (equal). **Qiaohuang Zeng:** conceptualization (equal), funding acquisition (equal), supervision (equal), writing – review and editing (lead).

## Ethics Statement

This study was approved by the Institutional Animal Ethical Committee of Guangdong Provincial Second Hospital of Traditional Chinese Medicine.

## Conflicts of Interest

The authors declare no conflicts of interest.

## Supporting information


**Figure S1:** fsn371064‐sup‐0001‐FigureS1.docx.

## Data Availability

Data supporting the findings of this study can be obtained upon request from the corresponding author.
