# Supplementary material for: Shikonin Ameliorates Alcohol‐Associated Liver Disease by Reducing Oxidative Stress, Apoptosis, and Inflammation
Source: Food Sci Nutr. 2025 Oct 8;13(10):e71064. doi: 10.1002/fsn3.71064 (PMC12507725; doi:10.1002/fsn3.71064)
Supplement: Supplementary file 1 — Figure S1: fsn371064‐sup‐0001‐FigureS1.docx. [file FSN3-13-e71064-s001.docx]

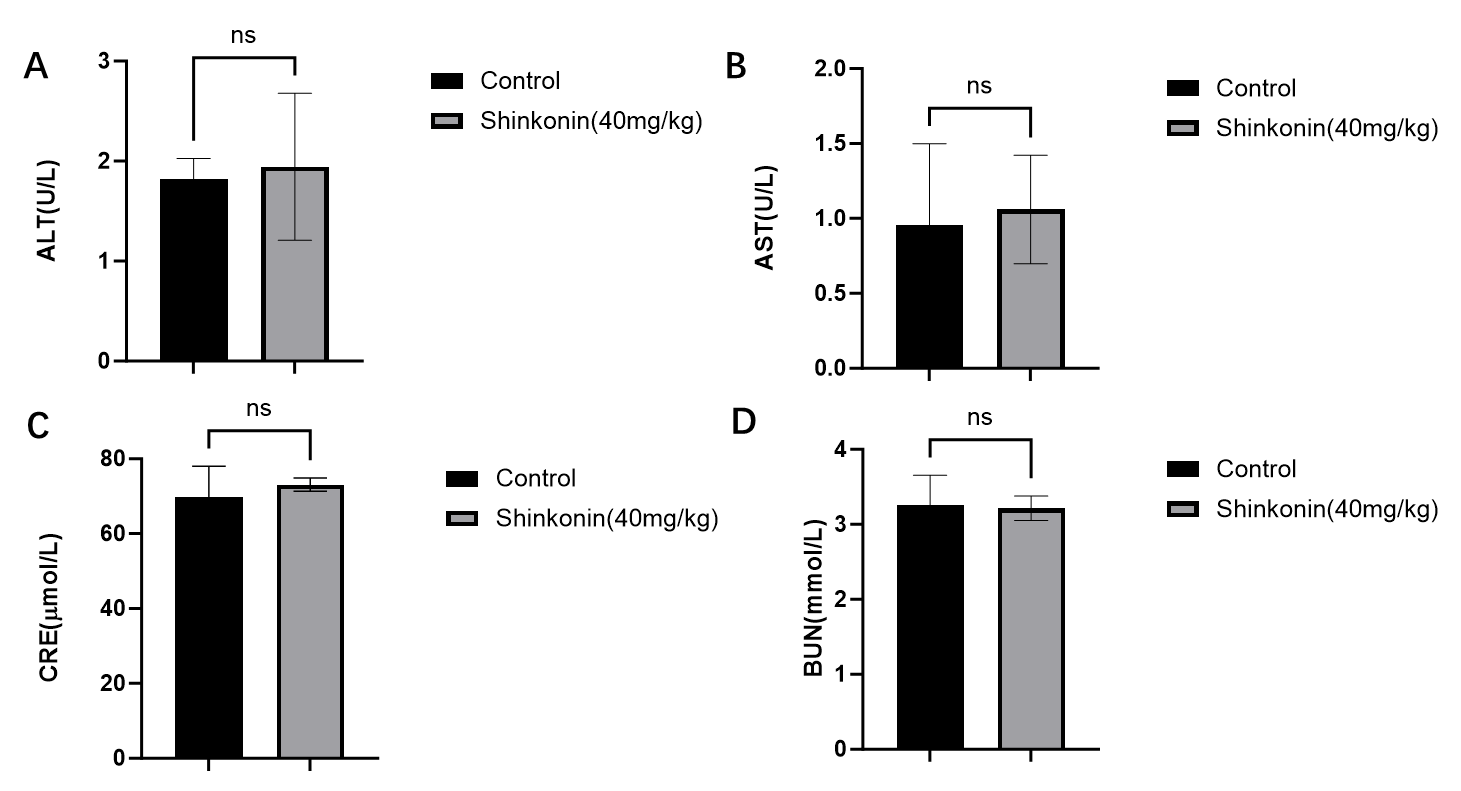


**Supplemental Figure 1 Evaluating the safety of Shikonin**. Six male Sprague-Dawley rats were randomized into control group and SKN-treated group (40 mg/kg body weight/day), n=3/group. Following 28 consecutive days of treatment. (A-D) There was no statistically significant difference in ALT, AST, CRE and BUN levels between the control group and SKN group of rats (all *p*>0.05). Data are expressed as mean ± SD (*n*=3),statistical analysis comprised independent-samples T test, ns *p*＞0.05.
